# Supplementary material for: Host-dependent resistance of Group A Streptococcus to sulfamethoxazole mediated by a horizontally-acquired reduced folate transporter
Source: Nat Commun. 2022 Nov 30;13:6557. doi: 10.1038/s41467-022-34243-3 (PMC9712650; doi:10.1038/s41467-022-34243-3)
Supplement: Supplementary file 1 — Supplementary Information [file 41467_2022_34243_MOESM1_ESM.pdf]

Supplementary Information

Host-dependent resistance of Group A *Streptococcus* to sulfamethoxazole mediated by a horizontally-acquired reduced folate transporter.

M. Kalindu D. Rodrigo, Aarti Saiganesh, Andrew J. Hayes, Alisha M. Wilson, Jack Anstey, Janessa L. Pickering, Jua Iwasaki, Jessica Hillas, Scott Winslow, Tabitha Woodman, Philipp Nitschke, Jake A. Lacey, Karen J. Breese, Mark P. G. van der Linden, Philip M. Giffard, Steven Y. C. Tong, Nicola Gray, Keith A. Stubbs, Jonathan R. Carapetis, Asha C. Bowen, Mark R. Davies, Timothy C. Barnett*

* Email: timothy.barnett@telethonkids.org.au

This PDF file includes:

Supplementary Note 1
Supplementary Methods
Figures 1 to 13
Tables 1 to 10

Supplementary Note 1

Metabolic profiling of MH-Bm and MH-Ox media. The differences in the susceptibility of TB08-2-14 to SXT on MHF-Ox and MHF-Bm media suggest that there are differences in the composition of MH media from these different suppliers. To investigate this, we first examined the composition of MH base medium from each supplier using nuclear magnetic resonance (NMR) spectroscopy. Using this approach, we found several differences in the composition of MH base medium from each supplier, with the MH-Ox medium containing higher levels of adenine, uracil and TRIS, while the MH-Bm medium contained higher levels of uridine, glucose and an unknown guanine moiety containing compound (Supplementary Fig. 11). However, we were unable to identify any major differences in folate pathway compounds using this approach. To further investigate the composition of MH-Ox and MH-Bm, we performed untargeted metabolomics analysis using reversed-phase LC-MS/MS. Confirming the results obtained with NMR spectroscopy (above), we observed multiple differences in the composition of each medium following positive and negative ionisation (Supplementary Fig. 12, Supplementary Table 10), but did not detect differences in folate pathway intermediates. Untargeted LC-MS profiling followed by multivariate statistical analysis (principal component analysis (PCA) and orthogonal projections to latent structures discriminant analysis (OPLS-DA) and database peak annotation revealed significant differences in a number of analytes (Supplementary Fig. 12), including higher levels of guanosine, uridine, methionine and peptides Pro-Ile-Ile and Pro-Val-Ile in the MH-Bm medium, with the MH-Ox medium containing higher levels of adenine, uracil, pyrrolidonecarboxylic acid and citric acid (Supplementary Table 10). Further comparison of spectra against reference standards for folate pathway intermediates revealed no significant differences other than a slight increase in 5,10-methylene-THF in MH-Bm (Supplementary Fig. 13). Thus, while there were major differences in the gross composition of MH media from each supplier, we were unable to identify substantial differences in the concentration of folic acid or reduced folate derivatives that might explain the relative performance of each media for detecting ThfT-mediated SMX resistance.

Supplementary Methods

^{1}H NMR sample preparation: The cell media were thawed at 6 °C for 2 h. 560 µL of the cell medium was directly injected into the NMR tube. Afterwards 40 µL of D_2O were added and the mixture was mixed thoroughly. All samples were prepared in standard 5 mm outer diameter NMR tubes.

^{1}H NMR spectroscopy data acquisition and processing parameters: NMR spectroscopic analyses were performed on a 600 MHz Bruker Avance III HD spectrometer, equipped with a 5 mm BBI probe and fitted with a Bruker SampleCase set to 6 °C. All spectra were acquired at 300 K and the correct temperature was ensured with a 99.8% Methanol-d_4 sample prior to measurement. The standard one-dimensional (1D) experiments with solvent suppression (pp: noesygppr1d) were acquired with 128 scans (+4 dummy scans), 64k data points, relaxation delay of 4.0 s, and a spectral width of 20 ppm resulting in a total experiment time of 14 min 53s. The spiking experiments were acquired with under the same conditions after addition of small quantities of the compound in question. Time domain data were Fourier transformed and processed manually using Bruker Topspin™ 3.6.2 or Bruker Topspin™ 4.1.3 to obtain phase and baseline corrected spectra. An exponential line broadening of 0.3 Hz was applied to the free induction decays (FID).

Standard preparation. Reference standards for each standard was from suppliers as indicated in Supplementary Table 6. Stock solutions were prepared at 1 mg/mL in DMSO, and further diluted to 10 µg/mL in methanol containing 0.1 % butylated hydroxytolunene (BHT) for LC-MS analysis.

Sample preparation. Media samples were filtered using a 3 kDa MWCO filter (Millipore Centriprep) and either directly injected for the detection of targeted tetrahydrofolic acid metabolites, or diluted 1 in 10 (v/v) with LC-MS grade water for untargeted profiling. Preparation was identical for both methods.

LC-MS method. Chromatographic separation was performed on a Waters Acquity I-class UPLC system (Waters, Wilmslow, UK), using a Waters Cortecs T3, 100mm x 2.1mm x 1.6 µm (Waters, Wilmslow, UK) kept at 40 ºC. Mobile phase A consisted of water containing 0.1 % formic acid, and mobile phase B was acetonitrile containing 0.1 % formic acid. The flow rate was set at 0.25 mL/min. Gradient elution was performed with initial conditions starting at 0 % B, held for 1.25 minutes, increasing to 10 % B at 6.00 minutes, 25 % B at 8.00 minutes and 75 % B at 10.50 minutes, followed by a wash step to 95 % B until 11.50 minutes. At 11.50 minutes, the flow was returned to initial conditions (0% B), allowing for 3.50 minutes of re-equilibration time. A 10 µL injection volume was performed. Mass spectrometry was performed on a Bruker Impact II QTOF-MS (Bruker, Bremen, Germany) with electrospray ionisation operated in positive and negative mode. The capillary voltage was set at 4500V in positive ionisation mode and 3500 V in negative ionisation mode. Drying gas was set at 10 L/min, gas temperature was 220 ºC, nebuliser pressure was 2.2 bar and the end plate offset was 500 V. MS1 scan rate was set at 12 Hz. Auto MS/MS was enabled, with 4 precursors automatically selected and data collected at a scan rate of 25 Hz per scan cycle, resulting in a total scan cycle time (MS1 + auto MS/MS) of 0.2 seconds. An internal calibration was performed by injection of 5 mM sodium formate solution in water:isopropanol (50:50 v/v) at the beginning of every run. Mass spectrometric data were collected with Compass HyStar 5.1 and O-TOF Control version 5.2. Data were reviewed using Compass DataAnalysis 5.2 (Bruker Daltonics, Bremen, Germany) and pre-processed in Metaboscape 2022 B. Multivariate statistical analysis was performed using SIMCA® 17.0 (Sartorius AG, Göttingen, Germany).

Supplementary Fig. 1.

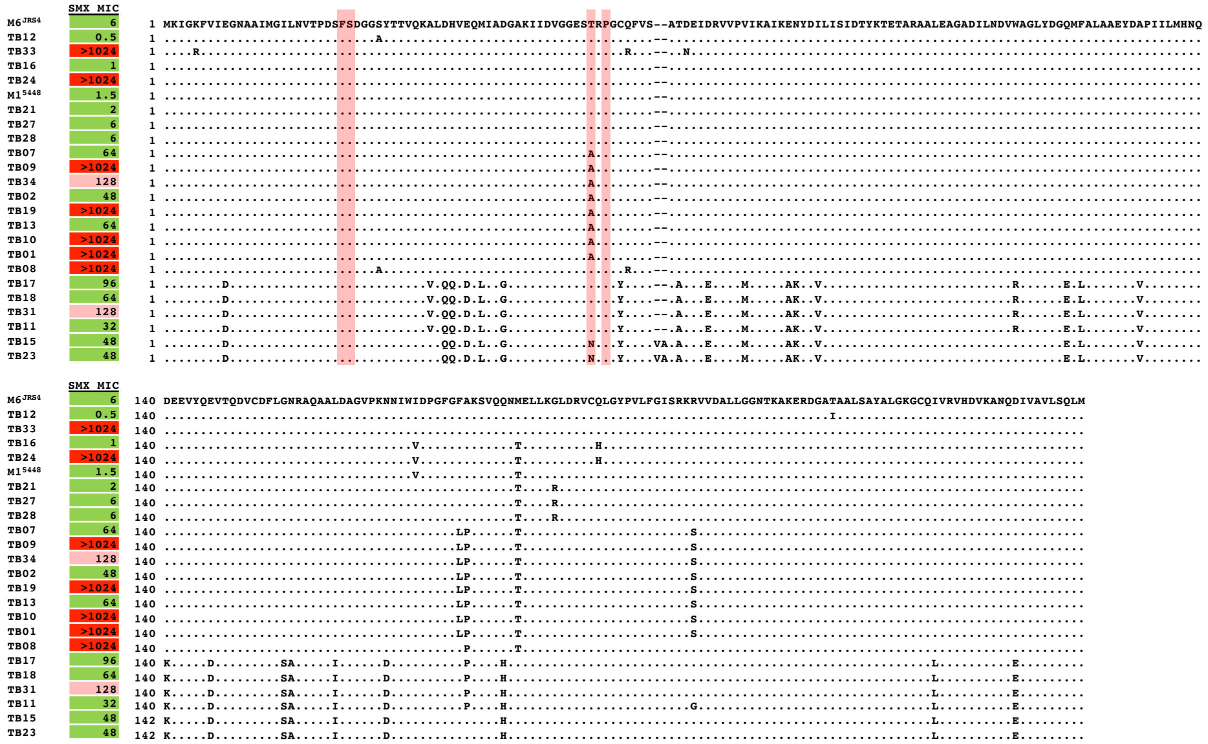

Amino acid sequence alignment of FolP and associated SMX MICs for GAS strains. Alignments were performed with CLUSATLW using translated *folP* gene sequences. Residues identified by structural[16] and biochemical[17] studies as being important for SMX resistance are highlighted in red. Genbank accessions were as follows: M6^{JRS4} (AKI76294.1), M1^{5448} (AKK70503.1). SMX MIC values are from Supplementary Table 1. Shading indicates level of sensitivity/resistance: Green, sensitive (SMX MIC <100 μg/ml); Light red, low resistance (SMX MIC 100-500 μg/ml); Dark red, highly resistant (SMX MIC >500 μg/ml).

Supplementary Fig. 2.

a

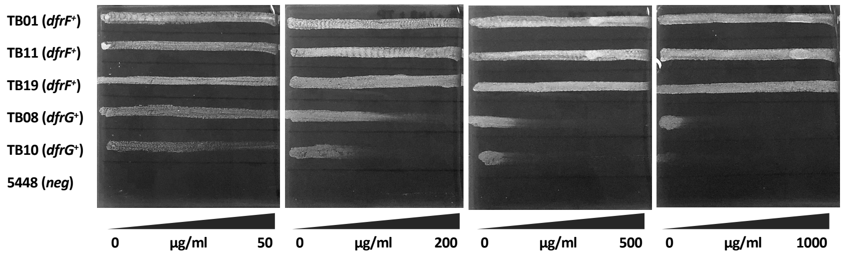

b

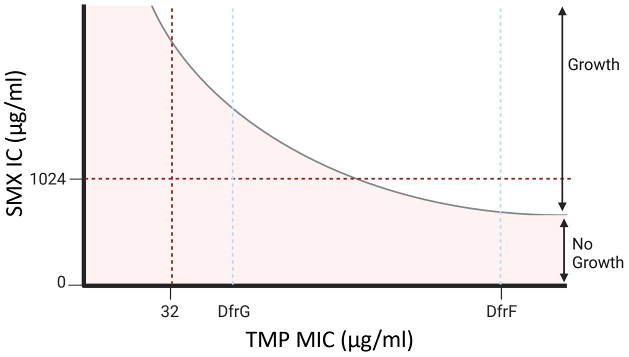

***dfrF* confers very high TMP-resistance.** (**a**) Comparison of TMP MICs for *dfrF*-positive and *dfrG*-positive GAS strains measured on gradient agar plates. The TMP gradient range is indicated below each image. *dfrF*-positive GAS strains exhibit higher TMP resistance than *dfrG*-positive GAS strains. (**b**) Schematic showing hypothetical effect of DfrF and DfrG on synergy of SMX and TMP. Limits of detection of SMX (1024 µg/ml) and TMP (32 µg/ml) Etest assays are indicated with red dashed lines. Relative TMP resistance provided by DfrG and DfrF are indicated with light blue dashed lines. The red zone below the synergy curve represents SXT inhibition of growth, while the white zone above the curve represents bacterial growth.

Supplementary Fig. 3.

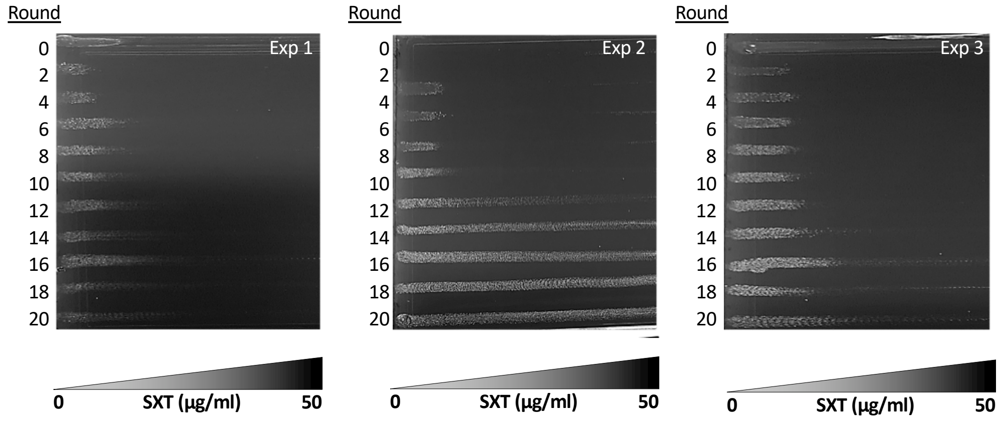

***In vitro* evolution of GAS strain TB08 on SXT gradient plates.** Growth of TB08 on SXT gradient plates following successive rounds of selection on SXT gradient plates. Each gradient plate demonstrates the bacterial growth following every second round of selection.

Supplementary Fig. 4.

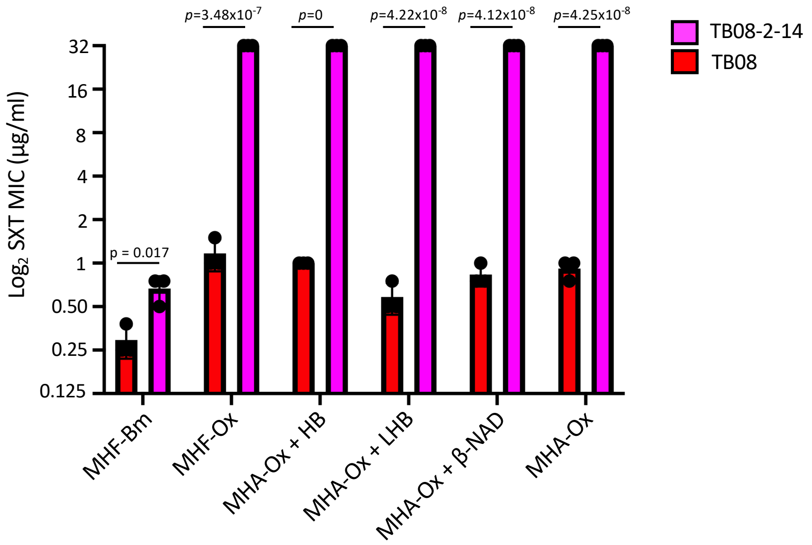

SXT Etest results of TB08 and TB08-2-14 (round 14, Exp 2) strains on different formulations of agar. Results are mean ± SD of three biological replicates. HB, 5% horse blood; LHB, 2.5% lysed horse blood. Differences assessed using a two-tailed, unpaired Student's t-test for MHF-Bm comparison. For all other comparisons, a one-sample *t*-test was used to determine differences from the maximum resolution of the Etest assay (32 µg/ml).

Supplementary Fig. 5.

```
                 ....:...10...:...20...:...30...:...40...:...50...:...60
FolT_Lcas     1 --MQVLSFSSPKLSTRNMVYMAMLMAMQIVLGRFSFGTP--WLKISPAFFATILMGYYFG
FolT_Lmes     1 MENTTRTWVFPKLDTRQFVLLAMLMALHMVLSRLTVGTN--VLQVSFAFVTMSLIAKWYG
ThfT_GAS      1 ---MSPIFNGPKMSVQRLVSIAMLIALTLVISKFSIPVIPQQLVISFAFIGNTMIGMIAG

                 ....:...70...:...80...:...90...:..100...:..110...:..120
FolT_Lcas    57 PWLAAGAAALNDQLSIMIFSPGANFPGFTISTAIAAMLYG-MFFHG-----KKVTVLRTL
FolT_Lmes    59 PLWSMLIAAILDVIGATIINPGAFFVGFTFTAMISALIYSLAYFKH-----DKTSWWRVS
ThfT_GAS     58 PIWAFISLALVDVTDNLASGSGNFIIWWTLMEAVQGLLYGFFFYRKPLSTANKKDWLYVS

                 ....:..130...:..140...:..150...:..160...:..170...:..180
FolT_Lcas   111 LAVGMVLLISNIILTTLWLNIMG------TPWQGIILPRTIKNVVMLPIQTALSYGTLK-
FolT_Lmes   114 VAVGLVLLIANIGLNSIWLVMMYHTAHDWPSFLAFVTPRVIKNLIMFPIQVGISYFLLNN
ThfT_GAS    118 LATIIIMLFGTFIFTPLLIQIYFH-----VPFWAQYAAGRWFKVFEIPLRVVITMLLVPA

                 ....:..190...
FolT_Lcas   164 -AIERIRPHL---
FolT_Lmes   174 QVISHTTKKIFS-
ThfT_GAS    173 LQRIPEIKKLNQA
```

Alignment of ThfT with FolT S component proteins from *Lacticaseibacillus casei* *Leuconostoc mesenteroides*. Alignment was performed using ClustalW, with identical residues shaded black and similar residues shaded grey. Lcas, *Lacticaseibacillus casei*; Lmes, *Leuconostoc mesenteroides*.

Supplementary Fig. 6.

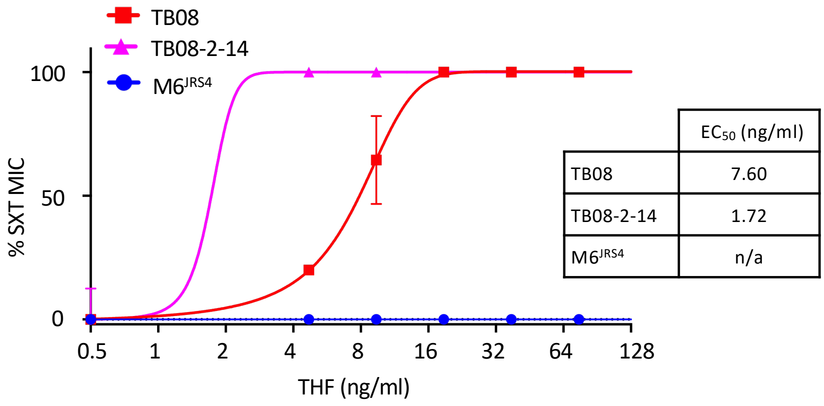

	EC_{50} (ng/ml)
TB08	7.60
TB08-2-14	1.72
$M6^{JRS4}$	n/a

ThfT confers SXT resistance in the presence of exogenous THF. Dose-response curves for the impact of exogenous THF on SXT resistance of $M6^{JRS4}$, TB08 and TB08-2-14 determined by broth microdilution. MICs are expressed as a percentage the maximum SXT concentration examined in this assay (50 µg/ml). Data from three biological replicates are presented as mean values ± SEM. EC_{50} values were calculated using Graphpad Prism software.

Supplementary Fig. 7.

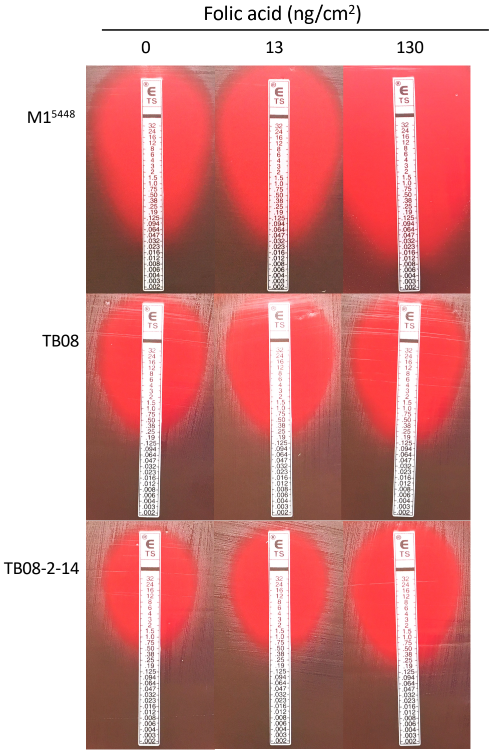

ThfT does not confer SXT resistance in the presence of exogenous folic acid. Susceptibility of GAS strains in the presence of exogenous folic acid as determined with Etest strips on MHF-Bm agar. Results are representative of two independent experiments.

Supplementary Fig. 8.

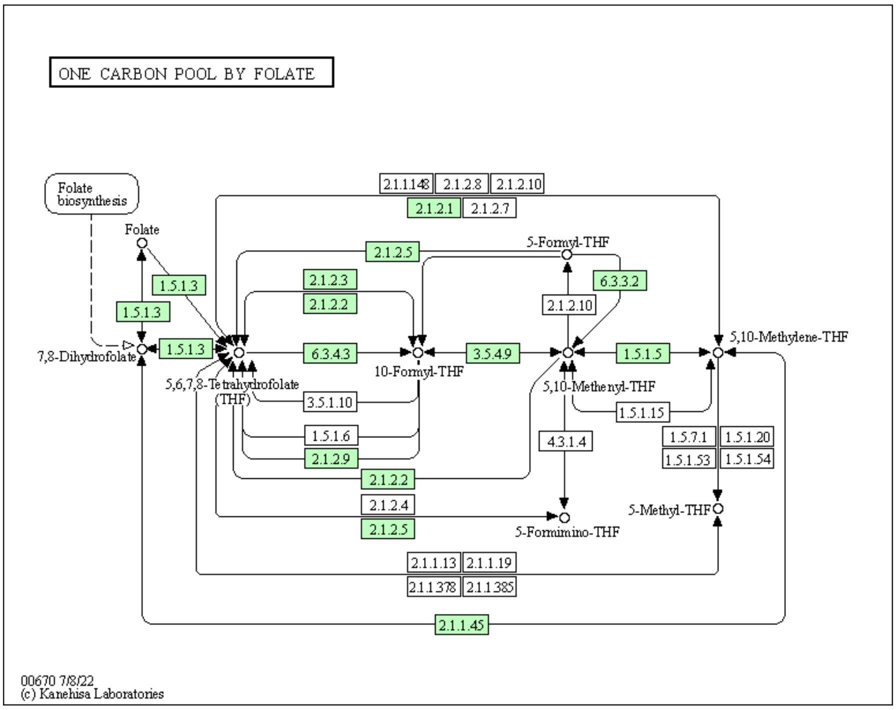

GAS proteins mapped onto the one carbon Kegg pathway map. Green boxes represent enzymes that have homologues in the GAS strain MGAS5005 genome. Image was downloaded from the KEGG database[43] (https://www.genome.jp/kegg-bin/show_pathway?spz00670) on 24 August 2022. Identical results are obtained with other GAS strains.

Supplementary Fig. 9.

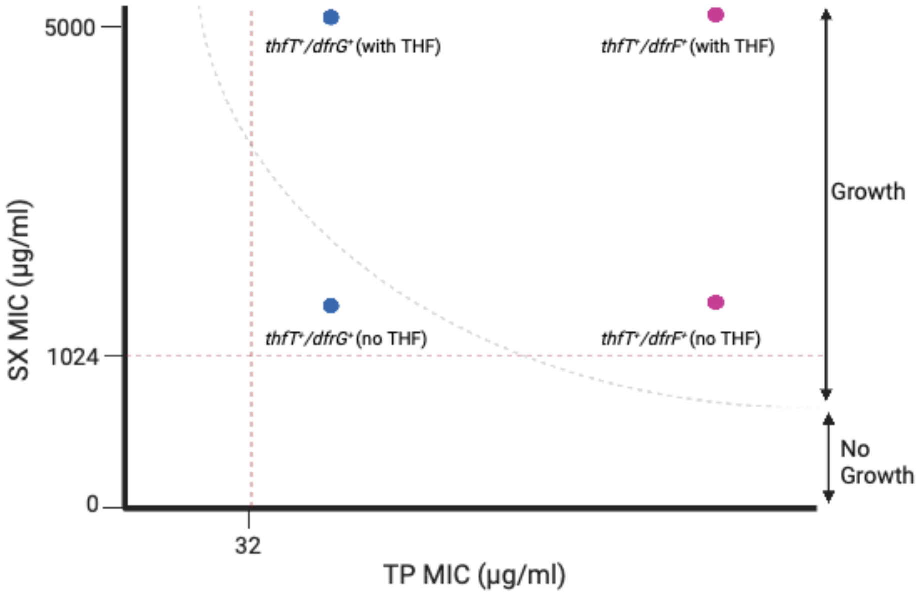

Schematic showing hypothetical effect of reduced folate compounds (e.g. THF) on synergy of SMX and TMP for GAS strains containing either *dfrG* (blue circles) or *dfrF* (magenta circles). Limits of detection of SMX (1024 µg/ml) and TMP (32 µg/ml) Etest assays are indicated with red dashed lines. The zone below the dashed curve represents SXT inhibition of growth, while zone above the curve represents bacterial growth.

Supplementary Fig. 10.

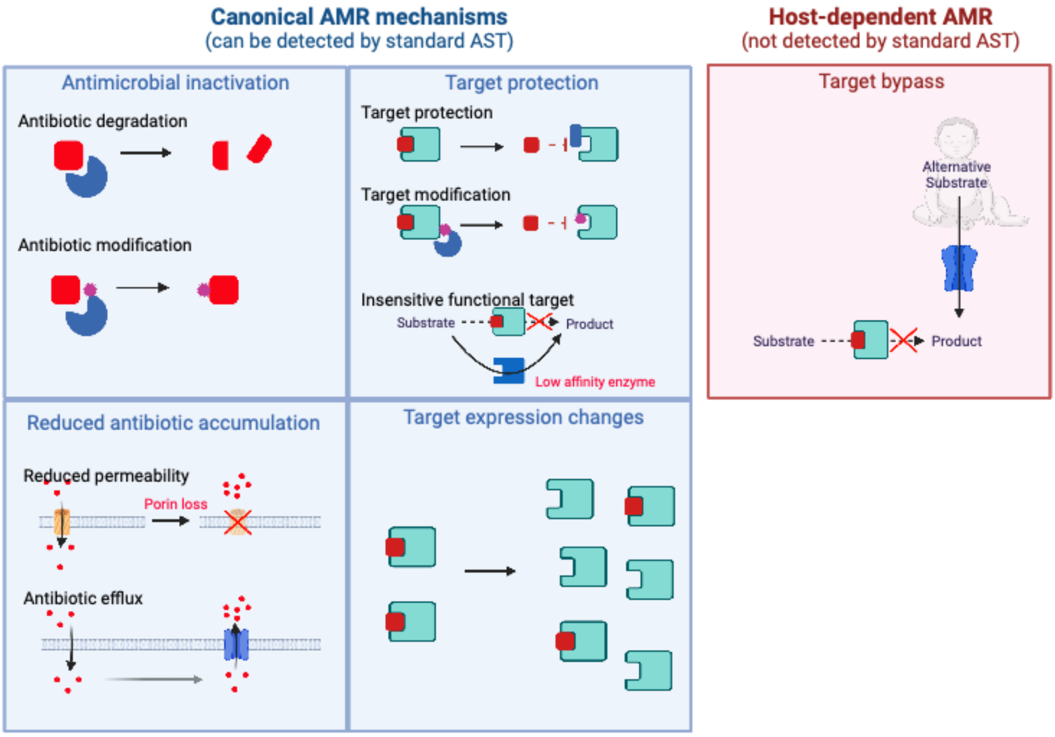

Overview of canonical antibiotic resistance mechanisms and comparison with SMX resistance mediated by ThfT. Red, antibiotic; Green, antibiotic target; Blue, AMR protein. Adapted from references *3-4*. Created with BioRender.com. AST, antibiotic susceptibility testing.

Supplementary Fig. 11.

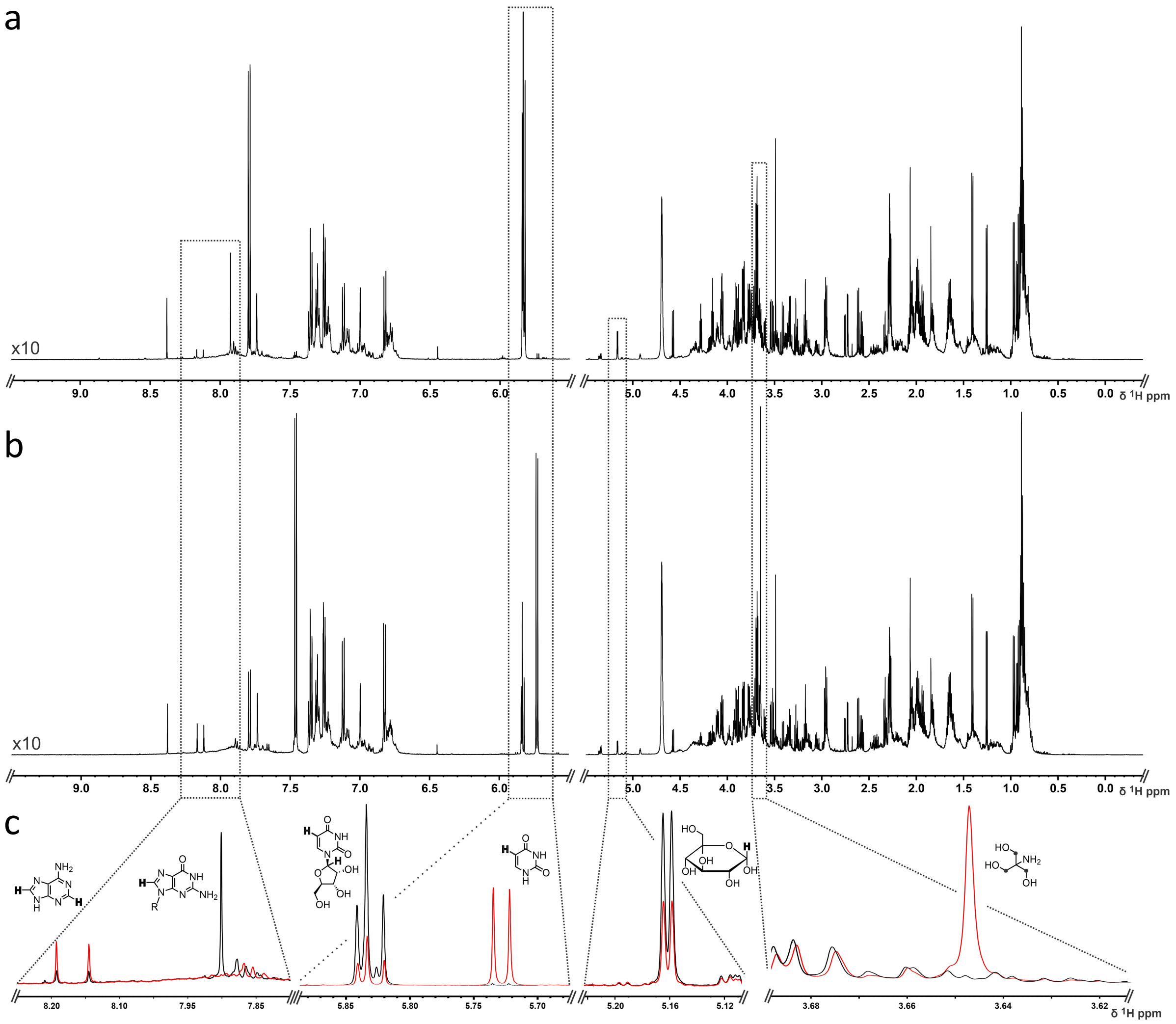

NMR spectroscopic measurements of MH-Ox and MH-Bm media. (**a**) 1D ^{1}H with solvent pre-saturation for the MH-Bm medium. The aromatic region (left) is scaled up by a factor of ten compared to the aliphatic region (right) of the spectrum. (**b**) 1D ^{1}H with solvent pre-saturation for the MH-Ox medium. The aromatic region (left) is scaled up by a factor of ten compared to the aliphatic region (right) of the spectrum. (**c**) Direct comparison of the two growth media MH-Bm (black) and MH-Ox (red) focussing on the proton NMR regions that showed major differences in peaks and/or peak intensities. Corresponding structures of the molecules or moieties are given next to the peak(s) in question and the proton which yields the peak is presented in bold. The structures (from left to right) are: Adenine, Guanine moiety containing compound (unknown), Uridine, Uracil, Glucose and TRIS.

Supplementary Fig. 12.

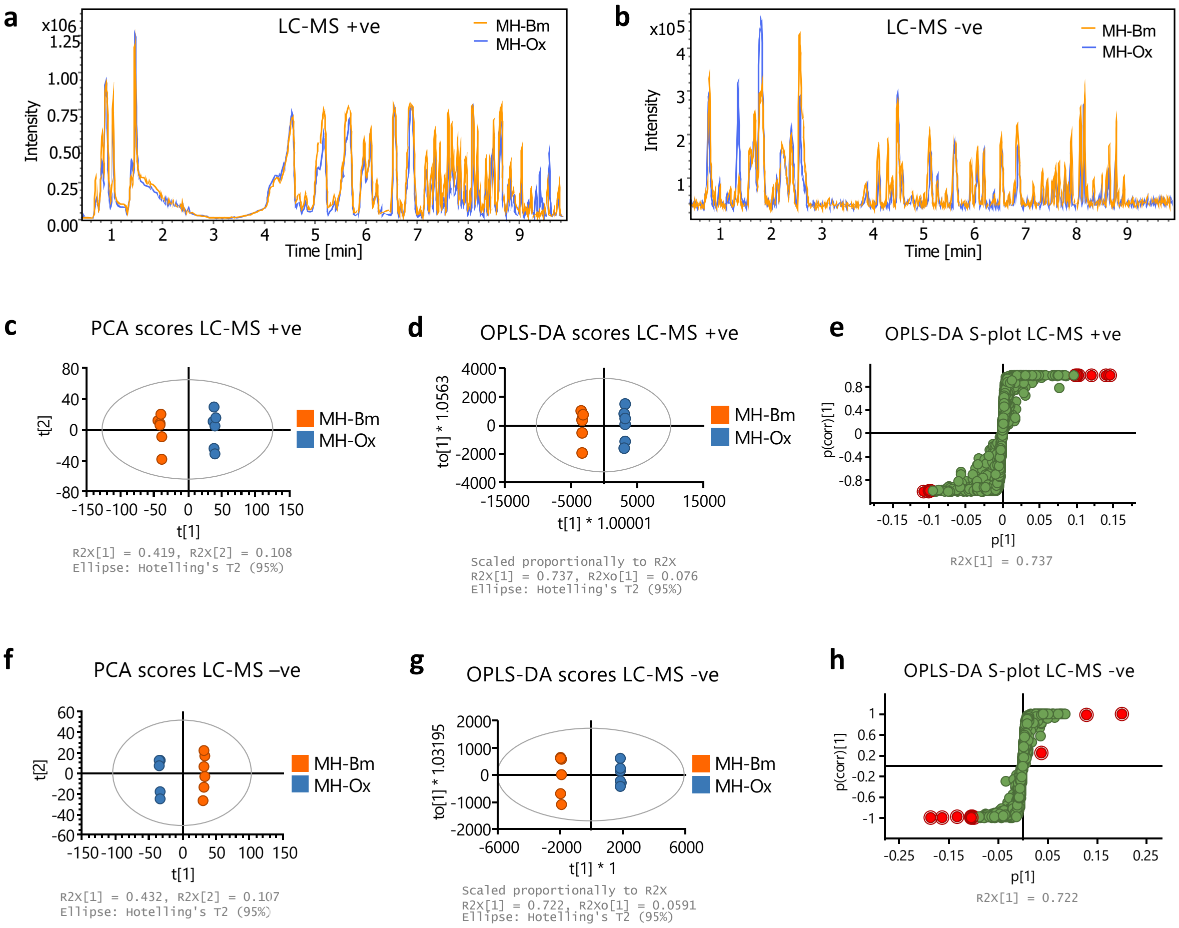

Untargeted LC-MS analysis of MH-Ox and MH-Bm media. (a) Comparison of the reversed-phase LC-MS base peak chromatograms in positive ionisation mode (MH-Bm orange, MH-Ox, blue). (**b**) Comparison of the reversed-phase LC-MS base peak chromatograms in negative ionisation mode (MH-Bm orange, MH-Ox, blue). (**c**) Principal component analysis (PCA) scores plot showing distinct separation resulting from replicate analysis of MH-Bm (orange) and MH-Ox (blue) media in LC-MS positive ionization mode. (**d**) Orthogonal projections to latent structures discriminant analysis (OPLS-DA) scores plot generated with data from LC-MS positive ionisation analysis of MH-Bm (orange) and MH-Ox (blue) media. (**e**) OPLS-DA S-plot generated from OPLS-DA of LC-MS positive ionisation data with the top 10 most discriminating features highlighted in red (Supplementary Table 10). (**f**) Principal component analysis (PCA) scores plot showing distinct separation resulting from replicate analysis of MH-Bm (orange) and MH-Ox (blue) media in LC-MS negative ionization mode. (**g**) Orthogonal projections to latent structures discriminant analysis (OPLS-DA) scores plot generated with data from LC-MS negative ionisation analysis of MH-Bm (orange) and MH-Ox (blue) media. (**h**) OPLS-DA S-plot generated from OPLS-DA of LC-MS negative ionisation data with the top 10 most discriminating features highlighted in red (Supplementary Table 10).

Supplementary Fig. 13.

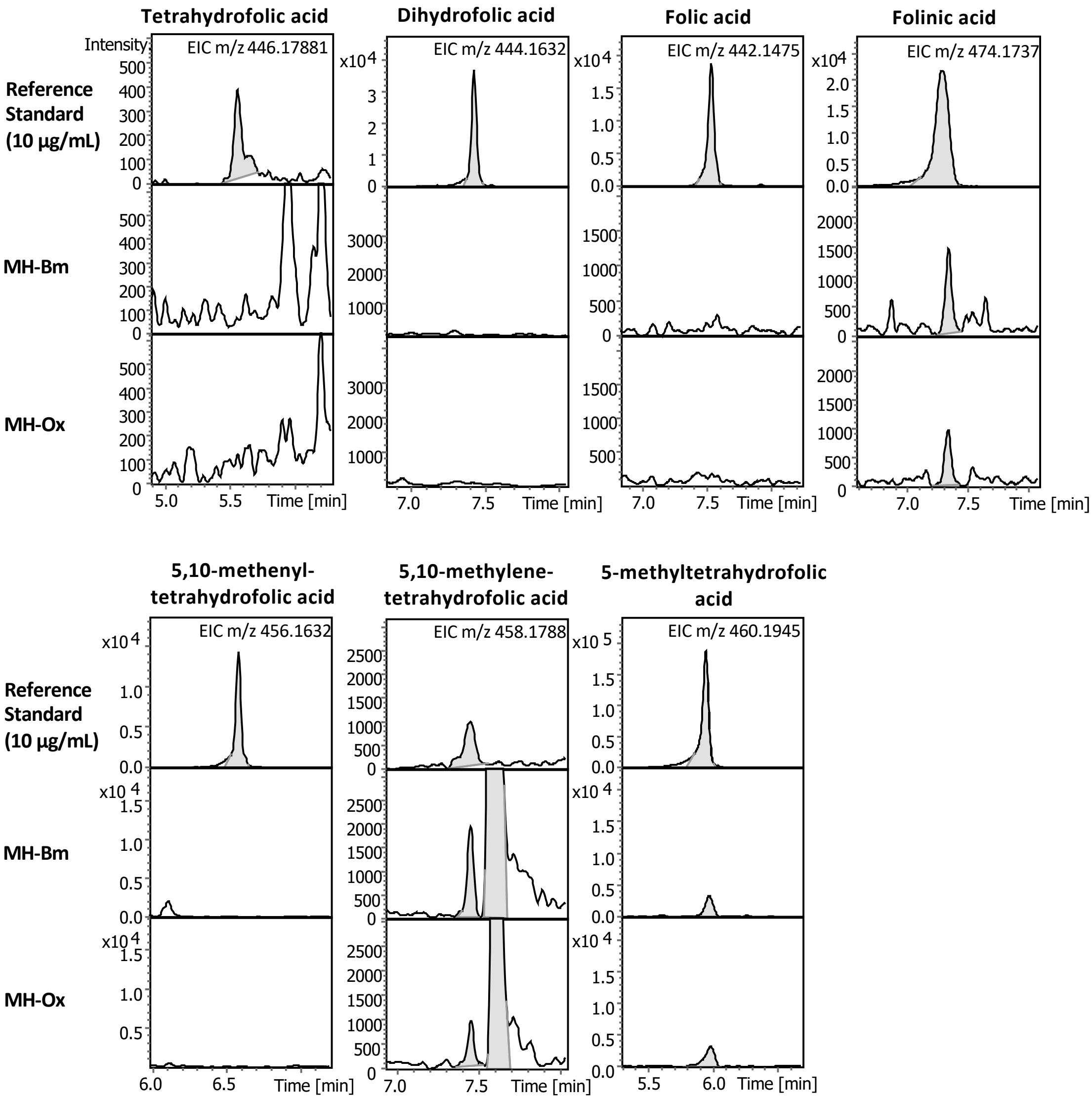

Targeted LC-MS analysis of folate pathway intermediates in MH-Ox and MH-Bm media. Extracted ion chromatograms (EIC) generated from reversed-phase LC-quadrupole-time-of-flight (QToF)-MS positive ionisation analysis of folate pathway intermediate reference standards at 10 µg/mL, MH-Bm and MH-Ox media.

Supplementary Table 1.

Strain	Lab ID*	*emmST*	MLST	Location	SMX	TMP	SXT	*dfrG*	*dfrF*	*dyr* I100L	*thfT*
M1^{5448}		1	28	USA	1.5	0.19	0.016				
M6^{JRS4}		6	37	USA	6	0.125	0.016				
TB01	838	49.4	534	India	>1024	>32	>32		■		
TB02	894	102.2	489	India	48	6	0.38			■	
TB07	981	102.2	349	India	64	4	0.25			■	
TB08	1001	4.2	289	India	>1024	>32	0.19	■			■
TB09	1002	49	371	India	>1024	>32	4		■		
TB10	1017	44	351	India	>1024	>32	1.5	■			■
TB11	1020	11	293	India	32	>32	0.5		■		
TB12	1024	22.8	360	India	0.5	>32	0.004		■		■
TB13	1033	102.2	349	India	64	1.5	0.38			■	
TB15	1380	113	677	India	48	1	0.125				
TB16	6653	28	458	Germany	1	>32	0.047		■		
TB17	6666	81	290	Germany	96	>32	0.5		■		
TB18	6679	81.2	290	Germany	64	>32	0.38		■		
TB19	7794	st854	-**	Germany	>1024	>32	>32		■		
TB21	8970	3	406	Germany	2	0.5	0.012				
TB23	9116	113	677	Germany	48	2	0.19				
TB24	9118	76	378	Germany	>1024	2	0.047				■
TB27	9386	3	15	Germany	6	2	0.032				
TB28	9395	3	15	Germany	6	2	0.047				
TB31	10009	49	840	Germany	128	>32	2		■		
TB33	10964	27	1163	Germany	>1024	1	0.032				■
TB34	10967	28	371	Germany	128	>32	2		■		

Susceptibility of GAS strains to TMP, SMX and SXT. Control strains M1^{5448} and M6^{JRS4} are included for comparison. MICs to each antibiotic was determined by determined with Etest strips on MHF-Bm agar. Each MIC value is the median value from three biological replicates. Source data are provided as a Source Data file. Shading indicates level of sensitivity/resistance: Green, sensitive (SMX MIC <100 µg/ml; TMP/SXT MIC <2 µg/ml); Light red, low resistance (SMX MIC 100-500 µg/ml; TMP/SXT MIC 2.0-10 µg/ml); Dark red, highly resistant (SMX MIC >500 µg/ml; TMP/SXT MIC >10 µg/ml). Presence (black) and absence (white) of TMP resistance genes in genome sequences is indicated to the right, as determined by BLASTN. *emmST*, *emm* gene sequence type. MLST, multi-locus sequence type. * Isolates from reference 13 (TB01-TB15) and reference 15 (TB16-TB34). ** TB19 does not belong to any currently-annotated MLST.

Supplementary Table 2.

	SXT MIC (μg/ml)		
THF:	0 ng/cm2	50 ng/cm2	
$M6^{JRS4}$	0.047 ± 0.0087	0.032 ± 0.0087	p=0.519
TB08 (T71; *dfrG*$^{+}$)	0.25 ± 0.072	>32	p=4.96x10^{-9}
TB10 (T71; *dfrG*$^{+}$)	1.5 ± 0.29	>32	p=3.66x10^{-7}
TB12 (T71; *dfrF*$^{+}$)	0.008 ± 0.0012	>32	p=2.00x10^{-14}
TB24 (V71)	0.023 ± 0.0052	0.125 ± 0.0017	p=0.00118
TB33 (V71)	0.032 ± 0.0087	0.125 ± 0.00	p=6.12x10^{-5}

Effect of exogenous THF on the susceptibility of GAS strains to SXT. Control strain $M6^{JRS4}$ is included for comparison. MICs to each antibiotic was determined by determined with Etest strips on MHF-Bm agar. Each MIC value is the mean value ± SD from three biological replicates. Source data are provided as a Source Data file. A two-tailed, unpaired Student's *t*-test was used for comparison of $M6^{JRS4}$, TB24 and TB33 experimental groups. For all other comparisons, a two-tailed, one-sample *t*-test was used to determine differences from the maximum resolution of the Etest assay (32 μg/ml).

Supplementary Table 3.

	SXT MIC + THF (ng/cm^2)			
Strain	0	200	300	400
TB08 (T71; *dfrG*$^+$)	0.125	>32	>32	>32
TB24 (A71)	0.047	0.50	0.75	0.75
TB33 (A71)	0.032	0.38	0.38	0.38

Susceptibility of TB08, TB24 and TB33 to SXT in the presence of different concentrations of exogenous THF. Results are median values (µg/ml) of three independent experiments determined with Etest strips on MHF-Bm agar. Source data are provided as a Source Data file.

Supplementary Table 4

Strain	MIC (µg/ml) no THF			MIC (µg/ml) with THF			*p* value		
	SMX	TMP	SXT	SMX	TMP	SXT	SMX	TMP	SXT
JRS4/vector	8.00 ± 0.00	0.273 ± 0.097	0.042 ± 0.009	7.33 ± 1.15	0.317 ± 0.110	0.034 ± 0.012	0.374	0.635	0.405
JRS4/P23-*thfT* (T71)	>1024	0.293 ± 0.075	0.058 ± 0.010	>1024	0.337 ± 0.075	0.105 ± 0.035	-	0.519	0.093
JRS4/ *thfT* (T71)	>1024	0.337 ± 0.075	0.058 ± 0.010	>1024	0.377 ± 0.125	0.094 ± 0.031	-	0.660	0.123
JRS4/P23-*thfT* (A71)	>1024	0.293 ± 0.075	0.068 ± 0.024	>1024	0.397 ± 0.179	0.094 ± 0.031	-	0.409	0.309
JRS4/ *thfT* (A71)	>1024	0.250 ± 0.000	0.058 ± 0.010	>1024	0.337 ± 0.075	0.105 ± 0.035	-	0.116	0.093

Mean, SD and *p* values for data in Fig. 4A. Values are mean ± SD for three biological replicates determined with Etest strips on MHF-Bm agar. A two-tailed, unpaired Student's *t*-test was used for comparison of groups.

Supplementary Table 5

	MIC (µg/ml) no THF			MIC (µg/ml) with THF			*p* value		
	SMX	TMP	SXT	SMX	TMP	SXT	SMX	TMP	SXT
NS5437	1.333 ± 0.289	0.250 ± 0.000	0.023 ± 0.000	1.667 ± 0.289	0.250 ± 0.000	0.023 ± 0.000	0.230	1.000	1.000
NS5437::*thfT*	3.667 ± 2.082	0.190 ± 0.000	0.023 ± 0.000	>1024	0.190 ± 0.000	0.105 ± 0.035	3.600×10^{-9}	1.000	1.408×10^{-3}

Mean, SD and *p* values for data in Fig. 4C. Values are mean ± SD for three biological replicates determined with Etest strips on MHF-Bm agar. A two-tailed, one-sample *t*-test was used to determine differences from the maximum resolution of the SMX Etest assay (1024 µg/ml) for NS5437::*thfT*. A two-tailed, unpaired Student's *t*-test was used for comparison of NS5437 groups.

Supplementary Table 6.

**Compound**	**Abbreviation**	**Supplier**	**Cat#**	**CAS#**
Tetrahydrofolic acid ($C_{19}H_{23}N_7O_6$)	THF	Sigma Aldrich	T3125	135-16-0
Dihydrofolic acid ($C_{19}H_{21}N_7O_6$)	DHF	Carbosynth	FD170209	4033-27-6
Folinic Acid ($C_{20}H_{23}N_7O_7$)	5-formyl-THF	Carbosynth	FF156776	58-05-9
10-Formyl-5,6,7,8-tetrahydrofolic acid disodium ($C_{20}H_{21}N_7Na_2O_7$)	10-formyl-THF	Carbosynth	FF168442	914800-65-0
(6R,S)-5,10-Methenyl-5,6,7,8-tetrahydrofolic acid ($C_{20}H_{21}N_7O_6$)	5,10-me$^+$-THF	Sapphire Bioscience	31333	7444-29-3
5, 10-Methylene-5,6,7,8-tetrahydrofolic acid ($C_{20}H_{23}N_7O_6$)	5,10-methyl-THF	Carbosynth	FM31365	3432-99-3
5-Methyltetrahydrofolic acid disodium salt ($C_{20}H_{23}N_7Na_2O_6$)	5-methyl-THF	Carbosynth	FM31095	68792-52-9
L-5-Methyltetrahydrofolate calcium ($C_{20}H_{23}CaN_7O_6$)	L-5-methyl-THF	Carbosynth	FM11406	151533-22-1

THF and related compounds used for metabolic rescue of GAS strains in the presence of antibiotics.

Supplementary Table 7

Compound	SMX MIC (µg/ml) NS5437	NS5437::*thfT*	*p* value
none	1.333 ± 0.289	2.333 ± 0.577	0.055
THF	1.500 ± 0.000	>1024	$1.19x10^{-12}$
DHF	0.917 ± 0.144	>1024	$1.19x10^{-12}$
5-formyl-THF	0.917 ± 0.144	>1024	$1.19x10^{-12}$
10-formyl-THF	0.917 ± 0.144	>1024	$1.19x10^{-12}$
5,10-me^{+}THF	0.917 ± 0.144	>1024	$1.19x10^{-12}$
5,10-methylene-THF	0.917 ± 0.144	>1024	$1.19x10^{-12}$
5-methyl-THF	1.167 ± 0.289	2.167 ± 0.764	0.101
L-5-methyl-THF	0.833 ± 0.144	2.667 ± 0.577	$5.94x10^{-3}$

Mean, SD and *p* values for data in Fig. 4A. Values are mean ± SD for three biological replicates determined with Etest strips on MHF-Bm agar. A two-tailed, one-sample *t*-test was used to determine differences from the maximum resolution of the SMX Etest assay (1024 µg/ml) for NS5437::*thfT*. For all other comparisons, a two-tailed, unpaired Student's *t*-test was used for comparison of groups.

Supplementary Table 8

Strain ID	*emmST*	MLST	Country	Year	Site	*thfT*	*dfrG*	*dfrF*	Accession
7368_6_94	86	ST4	Australia	2002	joint / synovial fluid	■			GCA_900991495
7368_8_77	86	ST4	Australia	1997	skin sore / abscess / burns / IV Site	■			GCA_900992295
19944_7_58	117	ST986	Brazil	2003	skin sore / abscess / burns / IV Site	■			GCA_900985075
SRR1104971	88	ST971	Canada	2010-2013	ND	■	■		SRR1104971
SRR1106008	74	ST120	Canada	2010-2013	ND	■	■		SRR1106008
19944_6_141	104	ST353	India	2007-2010	invasive	■			GCA_900983485
19944_6_145	80	ST701	India	2007-2010	Throat			■	GCA_900983565
19944_6_146	75	ST230	India	2007-2010	skin		■		GCA_900983525
19944_6_147	44	ST987	India	2007-2010	Throat	■		■	GCA_900983515
19944_6_148	22	ST360	India	2007-2010	skin	■		■	GCA_900983535
19944_6_154	44	ST178	India	2007-2010	Skin	■			GCA_900983615
19944_6_158	49	ST534	India	2007-2010	ND			■	GCA_900983655
19944_6_160	104	ST353	India	2007-2010	Throat	■			GCA_900983665
19944_7_4	75	ST357	India	2007-2010	Throat	■			GCA_900984895
19944_7_5	49	ST534	India	2007-2010	skin			■	GCA_900984955
19944_7_123	76	ST378	NZ	2013	Throat	■			GCA_900984075
19944_7_129	76	ST378	NZ	2013	Throat	■			GCA_900984115
19944_7_144	58	ST1004	NZ	2014	Throat			■	GCA_900984275
19944_7_145	76	ST378	NZ	2013	Throat	■			GCA_900984295
19944_8_45	76	ST378	NZ	2013	Blood	■			GCA_900994755
ERR1359618	58	ST985	UK	2014	ND	■		■	ERR1359618
ERR1359648	58	ST985	UK	2014	ND	■		■	ERR1359648
ERR1732468	11	ST293	UK	2014-2015	ND			■	ERR1732468
ERR1732551	74	ST120	UK	2014-2015	ND		■		ERR1732551
ERR1732614	49	ST371	UK	2014-2015	ND			■	ERR1732614
ERR1732992	76	ST378	UK	2014-2015	ND	■			ERR1732992
ERR1733243	76	ST378	UK	2014-2015	ND	■			ERR1733243
ERR1733400	218	ST292	UK	2014-2015	ND		■		ERR1733400
ERR1733501	49	ST228	UK	2014-2015	ND			■	ERR1733501
ERR1733622	49	ST371	UK	2014-2015	ND			■	ERR1733622
SRS2372037	82	ST896	USA	2015	ND			■	SRS2372037
SRS2372224	81	ST909	USA	2015	ND	■			SRS2372224

Carriage of *thfT*, *drfF* and *drfG* by GAS. Genome sequences from reference 27. Presence (black) and absence (white) of *thfT*, *drfF* and *drfG* in genome sequences was determined by BLAST.

Supplementary Table 9.

***S. dysgalactiae* (n=136)**	**Country**	**Year**	**Site**	***thfT***	***dfrF***	***dfrG***	**Accession**
DY107	China	2020	Cow		■		GCF_019856435
WCHSDSE-1	China	2013	Throat	■			GCF_001038445
UT_4277_BB	USA	-	Sterile	■			GCF_001682765
DB31752-13	Singapore	2013	Sterile	■			GCF_009650255

Carriage of *thfT*, *drfF* and *drfG* by *S. dysgalactiae*. Refseq genome sequences downloaded from NCBI on 12 August 2022. Presence (black) and absence (white) of *thfT*, *drfF* and *drfG* in genome sequences was determined by BLAST.

Supplementary Table 10.

	RT	m/z	Formula	Annotation	VIP	Higher
**Positive**	9.58	562.8210	C54H89N7O18		9.31045	MH-Ox
	8.46	326.7050	C28H45N17O2		8.88326	MH-Ox
	9.40	530.2970	C26H43NO10		7.69593	MH-Ox
	7.96	191.6260	C18H31N5O4		6.74514	MH-Bm
	8.10	385.2440	C18H32N4O5		6.56549	MH-Ox
	1.08	136.0620	C5H5N5	Adenine	6.41266	MH-Ox
	4.34	284.0990	C10H13N5O5	Guanosine	6.33763	MH-Bm
	9.32	379.7080	C34H47N17O4		6.29898	MH-Ox
	8.63	342.2380	C17H31N3O4	Pro Ile Ile	6.28548	MH-Bm
	8.17	328.2230	C16H29N3O4	Pro Val Ile	6.19526	MH-Bm
	**RT**	**m/z**	**Formula**	**Annotation**	**VIP**	**Higher**
**Negative**	1.41	111.0200	C4H4N2O2	Uracil	10.2991	MH-Ox
	2.61	487.1320	C18H34O9P2S		9.50986	MH-Bm
	4.34	282.0850	C10H13N5O5	Guanosine	8.31936	MH-Bm
	2.61	243.0620	C9H12N2O6	Uridine	6.79399	MH-Bm
	1.87	128.0350	C5H7NO3	Pyrrolidonecarboxylic acid	6.65050	MH-Ox
	8.09	243.1710	C12H24N2O3		5.33297	MH-Bm
	8.78	340.1880	C16H27N3O5		5.30046	MH-Bm
	2.72	457.1940	C19H30N4O9		5.26745	MH-Bm
	1.42	148.0440	C5H11NO2S	L-Methionine	5.04665	MH-Bm
	1.73	191.0200	C6H8O7	Citric acid	4.74788	MH-Ox

Discriminating features identified under positive and negative ionisation modes following untargeted LC-MS profiling of MH-Bm and MH-Ox media.
