# Supplementary material for: Host-dependent resistance of Group A Streptococcus to sulfamethoxazole mediated by a horizontally-acquired reduced folate transporter
Source: Nat Commun. 2022 Nov 30;13:6557. doi: 10.1038/s41467-022-34243-3 (PMC9712650; doi:10.1038/s41467-022-34243-3)
Supplement: Supplementary file 3 — Reporting Summary [file 41467_2022_34243_MOESM3_ESM.pdf]

nature portfolio

Corresponding author(s): Timothy Barnett

Last updated by author(s): Oct 12, 2022

Reporting Summary

Nature Portfolio wishes to improve the reproducibility of the work that we publish. This form provides structure for consistency and transparency in reporting. For further information on Nature Portfolio policies, see our Editorial Policies and the Editorial Policy Checklist.

Statistics

For all statistical analyses, confirm that the following items are present in the figure legend, table legend, main text, or Methods section.

n/a	Confirmed	
☐	☒	The exact sample size (*n*) for each experimental group/condition, given as a discrete number and unit of measurement
☐	☒	A statement on whether measurements were taken from distinct samples or whether the same sample was measured repeatedly
☐	☒	The statistical test(s) used AND whether they are one- or two-sided<br>*Only common tests should be described solely by name; describe more complex techniques in the Methods section.*
☒	☐	A description of all covariates tested
☒	☐	A description of any assumptions or corrections, such as tests of normality and adjustment for multiple comparisons
☐	☒	A full description of the statistical parameters including central tendency (e.g. means) or other basic estimates (e.g. regression coefficient) AND variation (e.g. standard deviation) or associated estimates of uncertainty (e.g. confidence intervals)
☐	☒	For null hypothesis testing, the test statistic (e.g. *F*, *t*, *r*) with confidence intervals, effect sizes, degrees of freedom and *P* value noted<br>*Give P values as exact values whenever suitable.*
☒	☐	For Bayesian analysis, information on the choice of priors and Markov chain Monte Carlo settings
☒	☐	For hierarchical and complex designs, identification of the appropriate level for tests and full reporting of outcomes
☒	☐	Estimates of effect sizes (e.g. Cohen's *d*, Pearson's *r*), indicating how they were calculated

Our web collection on statistics for biologists contains articles on many of the points above.

Software and code

Policy information about availability of computer code

Data collection

No software was used for data collection.

Data analysis

Data analysis was performed using the following software:
SKESA (version 2.2), available at https://github.com/ncbi/SKESA
Spades (version 1.09), available at https://github.com/ablab/spades
Prokka (version 1.14), available at https://github.com/tseemann/prokka
Abricate (version 0.8.2), available at https://github.com/tseemann/abricate
Breseq (version 0.35.1), available at https://github.com/barricklab/breseq
Screen Assembly (version 1), available at https://github.com/shimbalama/screen_assembly
CFX Manager (version 3.1), available at https://www.bio-rad.com/
Bruker Topspin (version 3.6.2 or 4.1.3), available at https://www.bruker.com/en/products-and-solutions/mr/nmr-software/topspin.html
Compass HyStar (version 5.1), available at https://www.bruker.com/en/products-and-solutions/mass-spectrometry/lc-ms/compass-hystar.html
O-TOF control (version 5.2), available at https://www.bruker.com/
Compass DataAnalysis (version 5.2), available at https://www.bruker.com/en/products-and-solutions/mass-spectrometry/ms-software/biopharma-compass.html
Metaboscape (version 2022 B), available at https://www.bruker.com/en/products-and-solutions/mass-spectrometry/ms-software/metaboscape.html
SIMCA (version 17.0), available at https://www.sartorius.com/en/products/process-analytical-technology/data-analytics-software/mvda-software/simca
Prism8 (version 9.3.1), available at https://www.graphpad.com/scientific-software/prism/

Microsoft Excel for Mac (version 16.55), available at https://www.microsoft.com/en-us/microsoft-365/excel
BioRender, available at https://app.biorender.com/

For manuscripts utilizing custom algorithms or software that are central to the research but not yet described in published literature, software must be made available to editors and reviewers. We strongly encourage code deposition in a community repository (e.g. GitHub). See the Nature Portfolio guidelines for submitting code & software for further information.

Data

Policy information about availability of data

All manuscripts must include a data availability statement. This statement should provide the following information, where applicable:

- Accession codes, unique identifiers, or web links for publicly available datasets
- A description of any restrictions on data availability
- For clinical datasets or third party data, please ensure that the statement adheres to our policy

The GAS whole genome sequencing data generated in this study have been deposited in the National Center for Biotechnology Information Sequence Read Archive database under accession code PRJNA791833 (https://www.ncbi.nlm.nih.gov/bioproject/PRJNA791833). The metabolomic profiling data generated in this study have been deposited in the Mendeley Data database under accession code h4tkyd6vwj (https://data.mendeley.com/datasets/h4tkyd6vwj). GAS genes mapped onto the One carbon pool by folate reference pathway was performed at the KEGG Pathway Database (https://www.kegg.jp/pathway/map00670). The processed source data generated in this study are provided in the Source Data file. All other data supporting the findings of this study are available from the corresponding author on reasonable request. Requests for materials should be addressed to T.C.B.

Human research participants

Policy information about studies involving human research participants and Sex and Gender in Research.

Reporting on sex and gender	N/A
Population characteristics	N/A
Recruitment	N/A
Ethics oversight	N/A

Note that full information on the approval of the study protocol must also be provided in the manuscript.

Field-specific reporting

Please select the one below that is the best fit for your research. If you are not sure, read the appropriate sections before making your selection.

☒ Life sciences ☐ Behavioural & social sciences ☐ Ecological, evolutionary & environmental sciences

For a reference copy of the document with all sections, see nature.com/documents/nr-reporting-summary-flat.pdf

Life sciences study design

All studies must disclose on these points even when the disclosure is negative.

Sample size	Sample sizes were chosen according to the standards in the field (PMID 30177740, PMID 33024089). At least 3 independent biological replicates for each condition. This generated sufficient data for statistical analysis.
Data exclusions	No data were excluded from the analyses.
Replication	Numbers of experimental replicates are stated in each figure legend. Reported results were consistently replicated across multiple experiments with all replicates generating similar results.
Randomization	No randomization was performed. Randomization is not applicable to the experimental design of the experiments performed in this study since each experiment was conducted by a single person.
Blinding	Investigators were not blinded. Blinding during analysis was not necessary because the results are quantitative and did not require subjective judgment or interpretation. Additionally, blinding was not possible since each experiment was conducted by a single person and sample labeling was required.

Reporting for specific materials, systems and methods

We require information from authors about some types of materials, experimental systems and methods used in many studies. Here, indicate whether each material, system or method listed is relevant to your study. If you are not sure if a list item applies to your research, read the appropriate section before selecting a response.

Materials & experimental systems

n/a	Involved in the study
☒	☐ Antibodies
☐	☒ Eukaryotic cell lines
☒	☐ Palaeontology and archaeology
☒	☐ Animals and other organisms
☒	☐ Clinical data
☒	☐ Dual use research of concern

Methods

n/a	Involved in the study
☒	☐ ChIP-seq
☒	☐ Flow cytometry
☒	☐ MRI-based neuroimaging

Eukaryotic cell lines

Policy information about cell lines and Sex and Gender in Research

Cell line source(s)	Human primary tonsil epithelial keratinocytes (lot# 10474) were obtained from Sciencell Research Laboratories (https://www.sciencellonline.com/human-tonsil-epithelial-cells.html).
Authentication	A certificate of analysis was obtained from the supplier. The cells were not authenticated.
Mycoplasma contamination	The cells were routinely tested for mycoplasma contamination. All cells used in this manuscript were negative.
Commonly misidentified lines (See ICLAC register)	No commonly misidentified cell lines were used in the study.
